# Generative Artificial Intelligence in Hip and Knee Arthroplasty: A Systematic Review of Emerging Clinical Applications in Patient Communication and Education, Documentation, and Decision Support

**DOI:** 10.2106/JBJS.OA.26.00077

**Published:** 2026-09-22

**Authors:** Ivan A. Garces, Andres G. Wong, Jonathan A. Brutti, Stefano A. Bini, Andrew McDaid, Charles M. Lawrie

**Affiliations:** 1 Herbert Wertheim College of Medicine, Florida International University, Miami, Florida; 2 Department of Orthopedic Surgery, University of California San Francisco, San Francisco, California; 3 Department of Mechanical & Mechatronics Engineering, University of Auckland, Auckland, New Zealand; 4 Baptist Health Orthopedic Care, Baptist Health South Florida, Miami, Florida

## Abstract

**Background:**

Generative artificial intelligence (AI), including large language models (LLMs), has been increasingly explored in orthopedic surgery; however, its application within total hip and knee arthroplasty (THA/TKA) has not been clearly characterized. Therefore, we performed a systematic review to further evaluate generative AI in THA/TKA across 3 clinical domains.

**Methods:**

A PubMed and Embase systematic literature review was performed on July 9, 2025, in accordance with the Preffered Reporting Items for Systematic Reviews and Meta-Analyses guidelines. Included studies evaluated generative AI use in THA/TKA and addressed 1 of our 3 domains. Excluded studies used nongenerative AI, involved populations not undergoing THA/TKA, or were non-English or non–peer-reviewed. Quality metrics that were assessed included blinded clinician ratings, readability scores, DISCERN scores, and diagnostic accuracy measures. The heterogeneity of the included studies led to a narrative synthesis, and no formal risk of bias was conducted.

**Results:**

Of the 91 articles retrieved, 23 met the inclusion criteria. ChatGPT versions 3.5 or 4 were assessed across all studies, and 3 studies included Google Gemini, Claude 3 Opus, and DeepSeek. Among studies in patient communication and education (n = 19), blinded clinician ratings showed that ChatGPT-generated responses to frequently asked questions (FAQ's) were as accurate, clear, and complete as surgeon-written responses. Regarding documentation (n = 2), LLMs demonstrated 97.5% to 100% accuracy in identifying operative report information and created patient consent documents with improved readability scores (grade level 12.6 vs. 16.8) and completeness (2.4/3 vs. 1.8/3). Among studies assessing decision support (n = 2), ChatGPT demonstrated high sensitivity when predicting surgical candidacy but low specificity when predicting surgical outcomes.

**Conclusion:**

LLMs performed well in several aspects of patient communication and education with support from high clinician ratings, accuracy, and readability scores. However, evidence for documentation and decision support remained limited. Moreover, proper implementation that considers clinical workflows could increase clinician efficiency and positively affect patient experience.

## Introduction

Total hip and knee arthroplasty (THA/TKA) procedures are expected to rise in the coming years^1^. In addition, orthopedic surgeons face significant time constraints because of burdensome electronic health record (EHR) note documentation, which can take up more than half of their workday, impair face-to-face time with patients, and contribute to clinician burnout^2^. Ambient artificial intelligence (AI) scribes have been used by providers to capture clinical notes automatically, but this study reviews applications of generative AI in THA/TKA.

Generative AI has been suggested as a tool to reduce repetitive clinical tasks; however, these tools can further remove patient–physician interaction. Large language models (LLMs) have already been implemented in general medicine to aid with note documentation, communication, and patients’ health literacy^3,4^. Orthopedic-specific uses include preoperative applications such as simplified educational resources and informed consent documents^5,6^; clinical applications in diagnostics, outcome prediction, and documentation^7^; and postoperative use for discharge instructions and patient questions^8^.

**Fig. 1:**
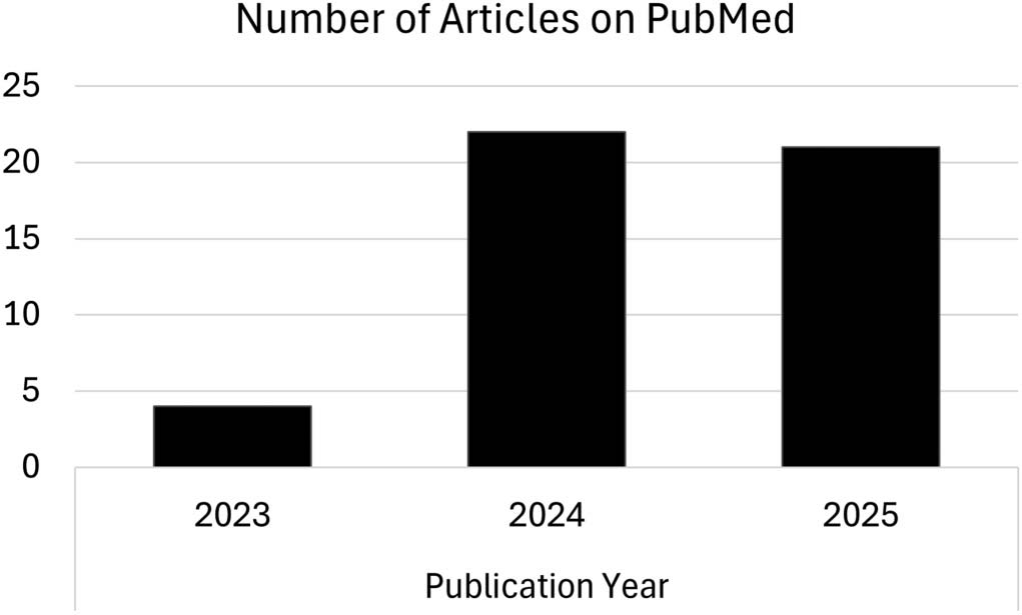
Bar chart depicting yearly PubMed-indexed publications using the search string: (“ChatGPT” OR “large language model” OR “generative AI”) AND (“hip arthroplasty” OR “knee arthroplasty” OR “THA OR TKA”). There were 4 articles in 2023, 22 in 2024, and 21 in 2025 (as of July). THA = total hip arthroplasty and TKA = total knee arthroplasty.

While the literature regarding the use of AI is increasing (Fig. 1), studies span different use cases without an overarching framework, and no systematic review has evaluated generative AI use in THA/TKA specifically. In this study, we systematically review the literature across 3 domains: patient communication and education, clinical documentation, and clinical decision support, and aim to provide an overview of LLM applications to THA/TKA-related tasks.

## Methods

### Search Strategy

PubMed and Embase were systematically searched on July 9, 2025, to identify studies evaluating generative AI in total hip and knee arthroplasty (THA/TKA).

The final search string used was as follows:

(“hip arthroplasty” OR “knee arthroplasty” OR “THA” OR “TKA”) AND (“ChatGPT” OR “GPT-4” OR “GPT-3.5” OR “large language model” OR “LLM” OR “generative AI” OR “Gemini” OR “Bard” OR “Claude” OR “LLaMA” OR “Google AI” OR “Anthropic”) AND (“patient education” OR “informed consent” OR “clinical decision-making” OR “treatment planning” OR “operative notes” OR “surgical documentation” OR “AI-generated documentation” OR “clinical documentation” OR “discharge instruction” OR “shared decision-making” OR “preoperative planning” OR “frequently asked questions” OR “FAQ” OR “minimum clinically important difference” OR “MCID” OR “outcome prediction” OR “patient questions”).

### Eligibility Criteria

**Fig. 2:**
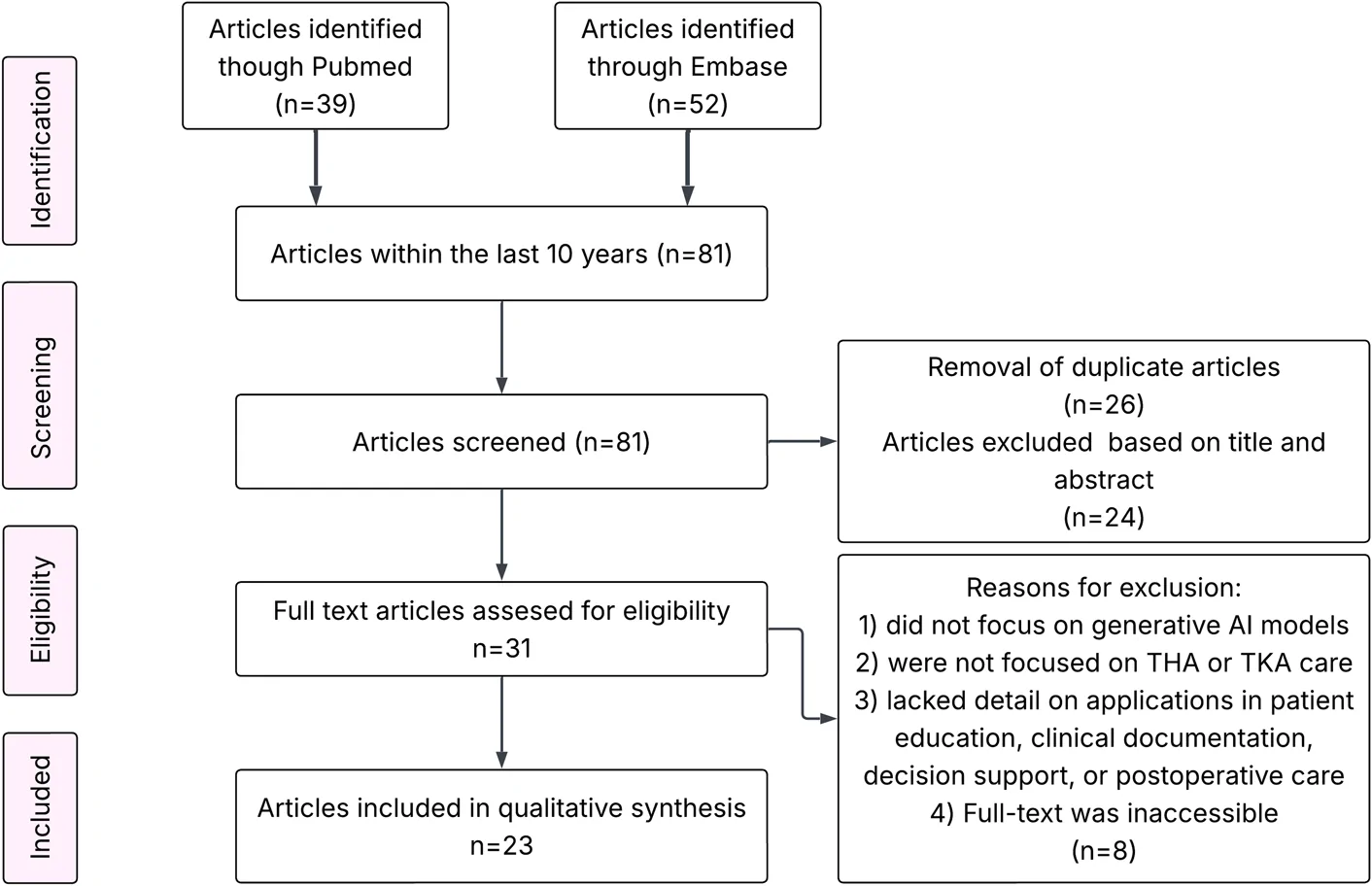
Flow diagram illustrating the study selection process for this systematic review. THA = total hip arthroplasty and TKA = total knee arthroplasty.

Included studies evaluated generative AI models such as ChatGPT or similar LLMs used in THA or TKA and in one of the 3 aforementioned domains. In addition, only English-language, peer-reviewed studies published from 2015 to 2025 were included to ensure consistency in data extraction and in methodological quality of the included studies. The exclusion criteria are provided in Figure 2. In addition, no other sources, reference list, or gray literature sources were used.

### Study Selection

Titles and abstracts were screened by 2 reviewers followed by independent full-text article review for eligibility in accordance with Preffered Reporting Items for Systematic Reviews and Meta-Analyses 2020 guidelines (Appendix 1). Any disagreements were resolved through discussion and consensus. Included studies were then grouped by category/domain, and the respective extracted data points are provided in Appendix 2. Patient demographics were inconsistently reported and not included in our analysis.

### Synthesis Approach

We performed a narrative synthesis by grouping studies by domain and qualitatively assessing their results to determine the accuracy, clinical utility, and shortcomings of generative AI in THA/TKA. Quantitative data were included from each of the respective studies as well as intraclass coefficients and confidence intervals, and no independent analysis was performed. Moreover, because of the heterogeneity of the included articles, no formal risk of bias, reporting bias, or certainty of evidence assessment was conducted.

## PROSPERO Registration

CRD420251089362.

## Protocol

The review protocol will be available on the PROSPERO database.

## Results

Of the 91 articles, 23 were included in this review. Included studies varied in designs, subjects, and goals, but all focused on ChatGPT. Evaluations included AI-generated responses, comparisons with clinician-authored responses, readability of the educational material, clinical decision support, and perception. Most articles (n = 19) were related to patient communication/education, two were related to clinical documentation, and two were related to clinical decision support. Some of the articles addressed additional orthopedic applications relevant to these procedures^9-11^.

Data assessment methods across studies included Likert scale ratings by expert raters, readability ratings, and other forms of statistical analysis. Furthermore, inter-rater reliability was measured with a moderate-to-high intraclass correlation coefficient in relevant studies. Finally, some studies evaluated response relevance (whether it answered the prompt), accuracy (whether it was factual according to guidelines), and completeness (whether it included important information). Other studies evaluated clarity (ease of reading), evidence-based content (providing data or citation sources), and consistency (similar response on requestioning).

### Patient Communication and Education

#### Accuracy and Clarity in Answering Patient FAQs

Magruder et al.^12^ found that ChatGPT's responses to 15 guideline-driven FAQs scored well, with relevance being the highest (4.43 ± 0.77), followed by clarity (4.22 ± 0.86), accuracy (4.10 ± 0.90), and consistency lowest (3.54 ± 1.10) when questioned again. In addition, Kienzle et al.^13^ observed that most DISCERN scores for preoperative TKA questions were greater than 3/5, despite 37% of references being fabricated or unverifiable. Moreover, ChatGPT-4 was able to respond to 12 periprosthetic joint infection FAQs, in which 7 responses required little revision, 4 needed moderate corrections, and 1 required none^14^.

When compared with clinicians, Liu et al.^15^ discovered that ChatGPT-4 responses to 10 TKA questions outperformed 3 of 5 attending surgeons in empathy, accuracy, completeness, and overall quality (4.4/5 vs. <4; p < 0.001). However, Smith et al.^11^ noted that experts had only more than partially agreed with ChatGPT's THA answers (2.25/3; 95% CI: 1.45-3.05) although specific explanations for agreement/disagreement were not explored.

Compared with other models, ChatGPT demonstrated significantly higher performance than DeepSeek in accuracy (8.7 ± 0.9 vs. 7.4 ± 1.2) and patient satisfaction (8.9 ± 0.8 vs. 7.6 ± 1.1) when answering questions^16^. In addition, Chen et al.^17^ showed that ChatGPT-4 provided more accurate and comprehensive answers than GPT-3.5, Google Gemini, and Claude 3 Opus when prompted as an orthopedic surgeon (accuracy = 3.73/5; comprehensiveness = 4.05/5; acceptability = 77.5%). Wright et al. found that 59.2% (95% CI: 50.0%-67.7%) of FAQ answers pertaining to THA/TKA were acceptable to at least 4 of 5 reviewers and further prompting of the model improved the reading level by 1 grade with minimal detail loss^18^.

With internet searches, Zhang et al.^19^ found that ChatGPT answered 88% of Google's top 50 People Also Ask questions about total knee replacement with 100% relevancy and high accuracy (4.9/5 and 4.6/5 for relevance and accuracy, respectively) when reviewed by orthopedic surgeons. Aydilek et al.^20^ found that ChatGPT had higher agreement with academic sources than Google (83.3% vs. 58.3%) and a higher DISCERN score (64.4 vs. 48.7). Finally, Dubin et al.^21^ found that only 25% of questions overlapped in information sourcing between Google and ChatGPT, with ChatGPT using government websites in 75% of responses compared with 65% commercial for Google.

#### Educational Materials, Anxiety, and Satisfaction

Kirchner et al.^9^ showed that TKA and THA pamphlets were able to be rewritten from a grade 12 to a grade 6 level, with Flesch Reading Ease scores increasing from 44 and 54 to about 77. In addition, Yüce et al.^22^ used ChatGPT-4 to simplify the text within 10 THA websites, with Hip Information Scoring System (HISS) scores improving from 9.5 to 21.5 after providing the model with HISS criteria. Taylor et al.^23^ tested ChatGPT's ability to answer 10 arthroplasty FAQs, which were blindly evaluated by 38 surgeons. ChatGPT's responses were recognized only 4 of 10 times, with over 90% of surgeons supporting its use to better serve diverse patient populations seeking online health information.

Gan et al.^24^ studied ChatGPT-assisted informed consent in TKA patients who received either traditional surgeon–led consent or physician-delivered ChatGPT answers. Patients receiving ChatGPT education had significantly lower perioperative anxiety on Hospital Anxiety and Depression Scale for Anxiety, Perioperative Apprehension Scale-7, and Visual Analogue Scale for Anxiety scales, along with greater satisfaction with education and hospital experience.

#### Postoperative Communication and Citation Concerns

In the study by Gencer et al.^25^ on ChatGPT-4's responses to sensitive questions regarding resumption of sexual intercourse after THA using 4 clinical scenarios and 24 patient queries, all the responses received high ratings for accuracy, comprehensiveness, and consistency (overall: 4.63/5) from the surgeons involved, with none being deemed unsafe. However, formal criteria scores were low (2.25/5 modified DISCERN, 1/5 Journal of the American Medical Association Benchmark Criteria), because of the lack of citations and qualifications. Similarly, assessment by Bains et al.^26^ of 60 questions regarding post-TKA operation included surgeon ratings of appropriateness at 73.3% for both ChatGPT and nurses' responses, with none of ChatGPT's answers “unreliable” in contrast to one by nurses. A total of 53.8% of patients preferred ChatGPT's response, whereas 34.0% opted for that of nurses, and 92.1% were unsure about trusting AI in this context.

Similarly, analysis by Dubin et al.^27^ revealed that 95% of ChatGPT-3.5's responses to 60 post-THA questions were rated as appropriate, compared with 93.3% of those from nurses. A total of 69.4% of patients preferred ChatGPT's answers, but 79.0% were uncertain about trusting AI. Moreover, Zhang et al.^19^ found that ChatGPT achieved 92.3% accuracy for recovery and 100% for activity questions although 1 weight-bearing response was inaccurate. Finally, Schwartzman et al.^28^ had ChatGPT cite peer-reviewed articles for 33 THA/TKA questions, finding only 35.8% of 109 references valid and 64.2% erroneous, although responses were scored as accurate and complete (4.4/6 and 1.92/3).

### Clinical Documentation

Attal et al.^29^ used ChatGPT-4 to extract critical information regarding fixation, technology, and approach of 240 THA reports using few-shot learning methods. High agreement was achieved (100% fixation, 98.9% technology, and 97.5% approach) and supplemented by concise rationales, where 87% of the justifications were consistent with the text.

In another study, Decker et al.^10^ assessed the performance of ChatGPT-3.5 at drafting risk-benefit-alternative (RBA) consent statements for operations such as TKA. AI-generated consent documents were better in readability and content than the documents prepared by surgeons (grade level of reading 12.6 vs. 16.8), having a higher degree of completeness and accuracy (2.4/3 vs. 1.8/3) and detailed information regarding benefits (2.8 vs. 1.5) and alternatives (3.0 vs. 1.3).

### Clinical Decision Support

Musbahi et al.^30^ evaluated ChatGPT-3.5's ability to recommend unicompartmental knee replacement or total knee replacement surgery among 32 cases, which were then compared with the consensus of 73 surgeons. ChatGPT showed 84% accuracy (95% CI: 0.67-0.94), 91% sensitivity (95% CI: 0.72-0.98), and 70% specificity (95% CI: 0.39-0.93) for unicompartmental knee replacement, with substantial agreement (Cohen’s κ = 0.63).

Zalikha et al.^31^ assessed the potential use of ChatGPT-3.5 in predicting outcomes after TKA based on unstructured clinical reports. In an 80-patient sample, ChatGPT detected most of the patients who improved (sensitivity 97.4%) but incorrectly predicted those who did not improve (specificity 33.3%). Compared with surgeons’ sensitivity of 90% and specificity of 63%, ChatGPT showed 65% accuracy, whereas surgeons scored 76%.

## Discussion

This systematic review evaluated the existing literature concerning the application of generative AI, with a focus on LLMs like ChatGPT, in THA and TKA. The primary takeaway is the potential utility of generative AI in the 3 domains, which could help address increasing patient numbers, documentation burden, and complex clinical decisions. Although expert opinion regarding AI-generated outputs is positive, patient trust remains a concern^26,27^. Health Insurance Portability and Accountability Act (HIPAA) compliance is also a concern as most LLMs are not certified compliant.

In communication and documentation in different healthcare scenarios, ChatGPT was able to generate responses that helped produce clear discharge summaries and clinical letters across different fields^32,33^, but occasional “hallucinations” still require oversight. Moreover, our review found that AI-generated responses were similar to clinician responses in clarity, empathy, and indistinguishability, while also improving readability and maintaining accuracy^9,10,18^.

One useful application is supporting postoperative patients when healthcare is difficult to access after-hours or on weekends. ChatGPT has demonstrated its capabilities in providing accurate information ranging from returning to activities to sexual health issues^19,25^. It has also shown similar or better performance compared with nurses for surgical questions^26,27^. However, most patients were not fully confident relying on AI for medical information, which may reflect the novelty of AI, concerns about reduced human interaction, and skepticism toward its accuracy.

In documentation, ChatGPT-4 has cut down the note-writing time by 1 to 2 minutes per complicated case in an example in pediatric emergency medicine^34^ and reduced time pressure while improving the documentation quality in a rehabilitation setting^35^. These findings align with our observation that LLM’s can extract operative details and draft improved consent forms with limited errors^10,29^, further showing the tools’ value in reducing administrative workload.

AI’s use in decision support has been mixed as well. Although ChatGPT's suggestions regarding surgical candidacy correlated with consensus opinion among experts^30^, lack of psychosocial or complication-specific considerations made recommendations less specific^31^. Clinical decision-making involves elements that cannot be quantified and require the experience from a surgeon. Clinician acceptance of AI-generated recommendations remains limited as suggestions still require oversight because of concerns over bias, workflow integration, and patient safety^36,37^.

One major limitation is the rapid pace of AI improvement as previous results risk becoming outdated with each new model release. Furthermore, heterogeneity between studies, including differences in methodology, sample size, and outcome measures, restricts generalization of results. Most literature analyzed LLM performance through human scoring rather than clinical application, with only 3 of 23 articles analyzing LLMs apart from ChatGPT, making it difficult to draw meaningful comparisons between models. Our review also has several limitations related to our approach, including language limitations, lack of conference abstract and unpublished articles, inconsistent terminology, and under-representation of HIPAA-compliant models. Moreover, the current publication landscape may also be influenced by bias toward publication of articles that report positive AI performance. In addition, hallucinations and citation fabrication are concerning, with 1 article reporting 37% fabricated or unverifiable citations^13^ while another reporting 64% errors^28^. Although these issues may be more manageable in patient education where physicians review content, they raise greater concerns in clinical decision support where knowing the source of recommendations is important.

## Conclusion

In general, LLMs were capable of producing more readable and effective responses than those developed by clinicians, with similar quality yet greater clarity and better ability in consent documentation while maintaining accuracy. However, support for decision-making remains inconsistent and still needs supervision. Because of publication lag, the results indicate at least 1-year-old performance; nevertheless, with rapid improvements, these technologies might be used to answer common inquiries, automate documentation, and support decision-making in the near future. In addition, implementing these technologies into practice will require approaches for EHR integration, clinician triggers, interaction auditing, and HIPAA compliance. Newer AI-based models allowing source verification may help solve source fabrication, whereas prospective testing remains required.

## Funding

This research did not receive any specific grant from funding agencies in the public, commercial, or not-for-profit sectors.

## Author Contributions

Ivan A. Garces: Study design, data collection, data analysis, manuscript preparation. Andres G. Wong and Jonathan A. Brutti: Manuscript preparation. Stefano A. Bini: Study design, manuscript preparation. Andrew McDaid: Study design, data collection, manuscript preparation. Charles M. Lawrie: Study design, data collection, data analysis, manuscript preparation.

## Appendix

Supporting material provided by the authors is posted with the online version of this article as a data supplement at jbjs.org (https://links.lww.com/JBJSOA/B342; https://links.lww.com/JBJSOA/B343). This content was not copyedited or verified by JBJS.
